# POL5551, a novel and potent CXCR4 antagonist, enhances sensitivity to chemotherapy in pediatric ALL

**DOI:** 10.18632/oncotarget.5094

**Published:** 2015-09-03

**Authors:** Edward Allan R. Sison, Daniel Magoon, Li Li, Colleen E. Annesley, Barbara Romagnoli, Garry J. Douglas, Gerald Tuffin, Johann Zimmermann, Patrick Brown

**Affiliations:** ^1^ Pediatric Hematology/Oncology, Texas Children's Cancer and Hematology Centers, Baylor College of Medicine, Houston, TX, USA; ^2^ Oncology and Pediatrics, The Sidney Kimmel Comprehensive Cancer Center, Johns Hopkins University School of Medicine, Baltimore, MD, USA; ^3^ Polyphor, Ltd., Allschwil, Switzerland

**Keywords:** acute lymphoblastic leukemia, CXCR4, microenvironment, chemokines, pediatric

## Abstract

The importance of the cell surface receptor CXCR4 and the chemokine stromal cell-derived factor-1 (SDF-1/CXCL12) is well-established in normal and malignant hematopoiesis. The Protein Epitope Mimetic POL5551 is a novel and potent antagonist of CXCR4. POL5551 efficiently mobilizes hematopoietic stem and progenitor cells, but its effects in acute lymphoblastic leukemia (ALL) have not been reported. Here, we demonstrate that POL5551 is a potent antagonist of CXCR4 in pre-B and T cell ALL cell lines and pediatric ALL primary samples. POL5551 has activity at nanomolar concentrations in decreasing CXCR4 antibody binding, blocking SDF-1α-mediated phosphorylation of ERK1/2, inhibiting SDF-1α-induced chemotaxis, and reversing stromal-mediated protection from chemotherapy. POL5551 is significantly more effective at inhibiting CXCR4 antibody binding than the FDA-approved CXCR4 inhibitor plerixafor in ALL cell lines and primary samples. We also show that treatment with POL5551 *in vitro* and cytarabine +/− POL5551 *in vivo* modulates surface expression of adhesion molecules, findings that may guide the optimal clinical use of POL5551. Finally, we demonstrate that POL5551 increases sensitivity to cytarabine in a xenograft model of a high-risk pediatric ALL, infant *MLL-*rearranged (*MLL-*R) ALL. Therefore, disruption of the CXCR4/SDF-1 axis with POL5551 may improve outcomes in children with high-risk ALL.

## INTRODUCTION

ALL is the most common pediatric malignancy. Advances in the treatment of ALL, including the use of multi-agent chemotherapy, improvements in supportive care, and risk stratification, have led to event-free survival rates that are now approaching 90% [1]. However, children and young adults with high-risk and relapsed/refractory ALL continue to have suboptimal outcomes. [2] We and others have demonstrated that interaction with the bone marrow microenvironment is important in a variety of hematopoietic malignancies [3–10]. Specifically, interaction between the cell surface receptor CXCR4 and the chemokine SDF-1 (CXCL12) is critical in signaling between leukemic blasts and the bone marrow microenvironment [11–17]. We previously demonstrated that CXCR4 is an important mediator of chemotherapy resistance in pediatric ALL and acute myeloid leukemia (AML), and that treatment with the FDA-approved CXCR4 inhibitor plerixafor could reverse stromal protection and chemotherapy resistance [8–10]. Therefore, disruption of the CXCR4/SDF-1 axis is a rational means to target extrinsic survival mechanisms in ALL. The novel Protein Epitope Mimetic (PEM) POL5551 is a selective and potent antagonist of CXCR4. PEMs are medium sized, fully synthetic cyclic peptide-like molecules that mimic the two most relevant secondary structure motifs involved in protein-protein interactions: Δ-hairpins and α-helices [18]. Recent reports have demonstrated that treatment with POL5551 inhibits vascular accumulation of CXCR4-expressing smooth muscle cells [19] and that POL5551 is a potent and effective mobilizer of hematopoietic stem and progenitor cells [20]. We offer the first report of this novel CXCR4 antagonist in pediatric ALL. We demonstrate that POL5551 is a potent antagonist of surface CXCR4 in pediatric ALL cell lines and primary samples. POL5551 also decreases SDF-1α-mediated phosphorylation of ERK1/2, inhibits chemotaxis induced by SDF-1α, and reverses stroma-mediated chemotherapy resistance. In addition, surface expression of adhesion molecules is affected by *in vitro* and *in vivo* treatment. Finally, POL5551 enhances sensitivity to chemotherapy in a xenograft model of infant *MLL*-rearranged (*MLL-*R) ALL, a high-risk subtype of ALL. Therefore, interruption of leukemia-microenvironment signaling with POL5551 may prove to be an effective strategy in the treatment of pediatric ALL.

## RESULTS

### POL5551 is a potent antagonist of surface CXCR4 in pre-B and T cell ALL cell lines

**Figure 1:**
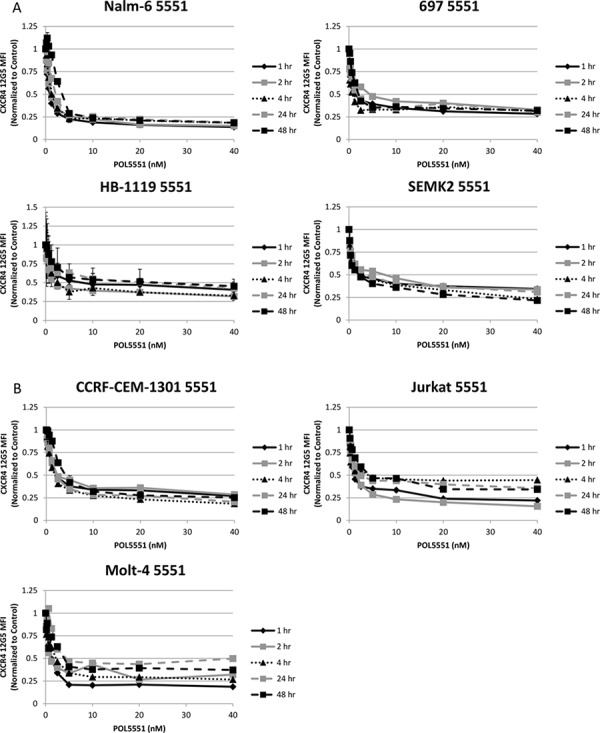

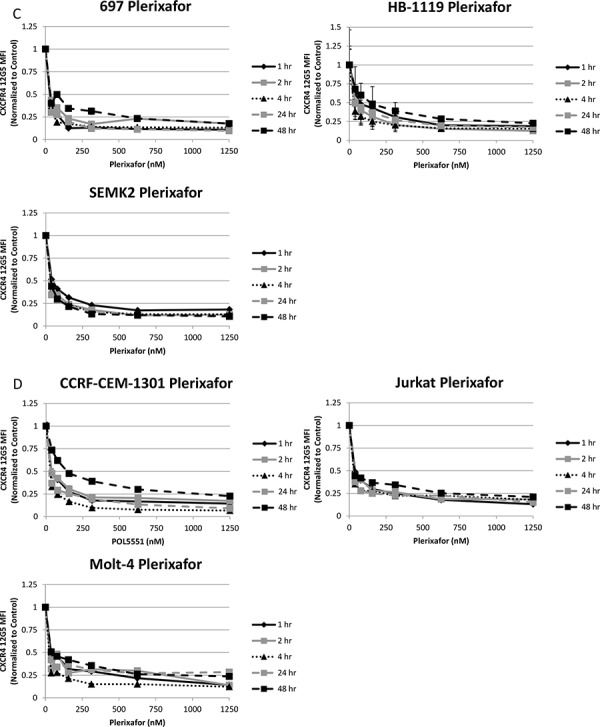

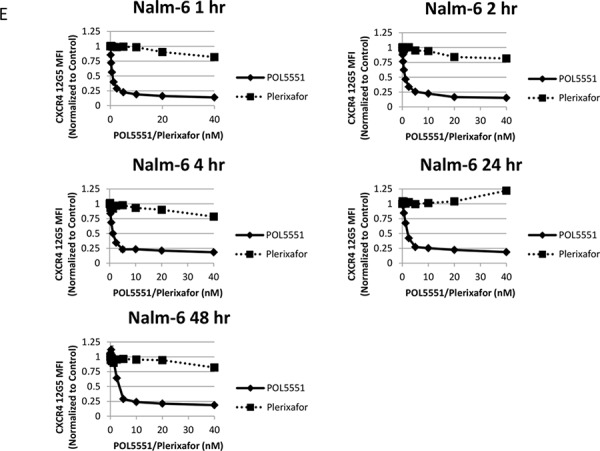
POL5551 potently inhibits binding of the 12G5 anti-CXCR4 antibody in ALL cell lines more effectively than plerixafor Cell lines were treated with a concentration range of POL5551 and plerixafor over a 48-hour time course. 12G5 anti-CXCR4 antibody binding was measured by FACS and MFI were normalized to control at each time point. Results after treatment with POL5551 in **A.** pre-B ALL cell lines and **B.** T ALL cell lines. Results after treatment with plerixafor in **C.** pre-B ALL cell lines and **D.** T ALL cell lines. **E.** Head-to-head comparison of POL5551 and plerixafor in Nalm-6. Error bars in this and subsequent figures represent the standard error of the mean (SEM).

We first wanted to determine if treatment with POL5551 could decrease antibody binding to surface CXCR4 in pediatric ALL. To do this, we treated a representative pre-B ALL cell line, Nalm-6, with a concentration range of POL5551 over a time course. We then harvested cells and measured binding of the 12G5 anti-CXCR4 antibody, which attaches to the SDF-1α binding site of CXCR4, by FACS as a marker for surface CXCR4 expression. Inhibition of 12G5 antibody binding by POL5551 was potent (IC50 of 12G5 binding at 1 hour 0.95 nM), rapid (<1 hour) and sustained (>48 hours) (Figure 1A). Next, we treated an additional 3 pre-B and 3 T cell ALL cell lines with POL5551 over a time course to determine if POL5551 could inhibit surface CXCR4 antibody binding across multiple cell lines. POL5551 potently antagonized surface CXCR4 antibody binding, as the IC50 of 12G5 binding was ≤2.5 nM at 1 hour in 5 of the 6 cell lines tested (Figures 1A–1B). In addition, 50% inhibition of surface CXCR4 antibody binding was maintained at 48 hours by treatment with ≤5 nM POL5551 in 5 of the 6 cell lines. For comparison, we also treated the cell lines with plerixafor and again measured 12G5 antibody binding. We found that while plerixafor also inhibited 12G5 antibody binding, POL5551 was significantly more potent, as several-fold higher concentrations of plerixafor were needed to achieve similar levels of reduction of 12G5 antibody binding (Figures 1C–1D). We verified these results by treating Nalm-6 with the same concentrations of POL5551 and plerixafor. We again found that POL5551 was able to consistently inhibit 12G5 antibody binding by 50% at concentrations <5 nM, while the maximum effect of plerixafor approached 20% inhibition at the maximum tested concentration of 40 nM (Figure 1E). From these experiments, we concluded that POL5551 is a potent and sustained antagonist of surface CXCR4 in pediatric ALL.

### POL5551 inhibits CXCR4 12G5 antibody binding but enhances 1D9 and 2B11 antibody binding

**Figure 2:**
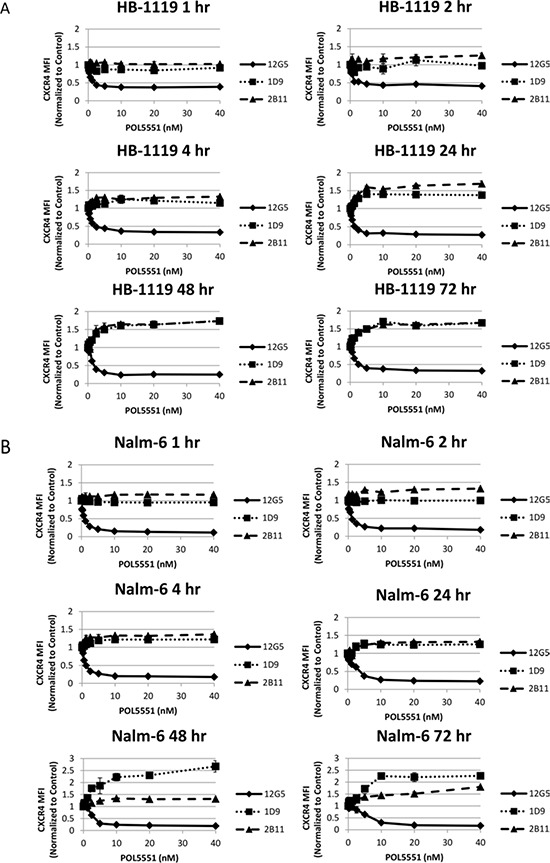

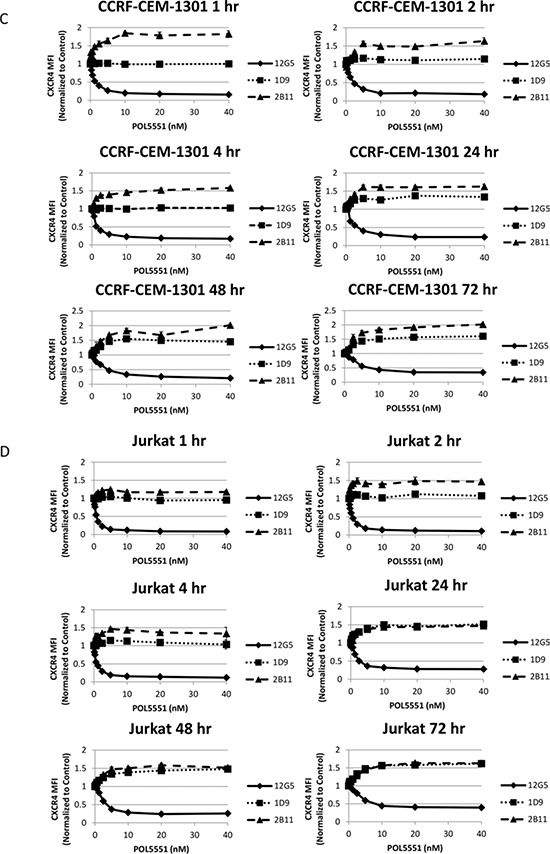
POL5551 simultaneously blocks 12G5 anti-CXCR4 antibody binding while increasing 1D9 and 2B11 anti-CXCR4 antibody binding Cell lines were treated with a concentration range of POL5551 over a 72-hour time course. Surface CXCR4 expression was measured by FACS using 3 different antibodies and MFI were normalized to control at each time point. All experiments were performed independently and in duplicate. Results after treatment with POL5551 in **A.** HB-1119, **B.** Nalm-6. All experiments were performed independently and in duplicate. **C.** CCRF-CEM-1301, and **D.** Jurkat.

We and others have shown that treatment of leukemic blasts with plerixafor leads to a decrease in 12G5 antibody binding to surface CXCR4, while simultaneously causing an increase in 1D9 antibody binding surface CXCR4 [10, 21]. While the 12G5 antibody competes with SDF-1α and plerixafor for the same CXCR4 binding site, [22] the 1D9 antibody binds to CXCR4 at an alternative site with which SDF-1α and plerixafor do not interact [21]. Therefore, 12G5 antibody binding can be interpreted as binding free surface CXCR4 that has not bound plerixafor or SDF-1α. Conversely, 1D9 antibody binding is a measure of total surface CXCR4. We sought to determine if the same phenomena occurred after treatment of ALL with POL5551. We treated 2 pre-B and 2 T cell ALL cell lines with a concentration range of POL5551 over 72 hours. At multiple time points, we measured binding to surface CXCR4 by flow cytometry using 3 anti-CXCR4 antibodies: 12G5, 1D9, and 2B11. The 2B11 anti-mouse CXCR4 antibody, which does not compete with SDF-1α or drug binding, was previously reported to bind to human CXCR4 [23], and we verified this in earlier experiments [10]. As in our previous experiments, 12G5 binding was decreased by POL5551 even at 1 hour and this effect was concentration-dependent. Notably, we found an increase in 1D9 and 2B11 antibody binding that was both time and concentration-dependent in all 4 cell lines tested (Figures 2A–2D). These results suggest that while POL5551 inhibits CXCR4 at the SDF-1α binding site, POL5551 leads to an increase in 1D9 and 2B11 binding to surface CXCR4 over time. Importantly, POL5551 is still able to potently block the SDF-1α-binding site through 72 hours of treatment.

### POL5551 induces increased surface expression of CXCR7, decreases SDF-1α-induced phosphorylation of ERK1/2, and inhibits SDF-1α-induced chemotaxis

**Figure 3:**
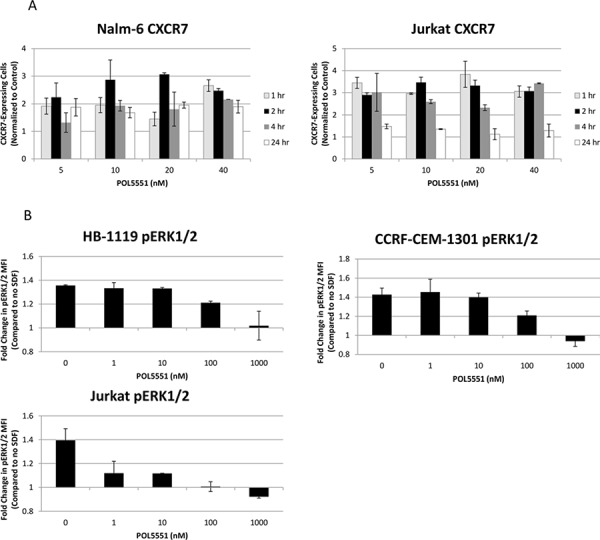

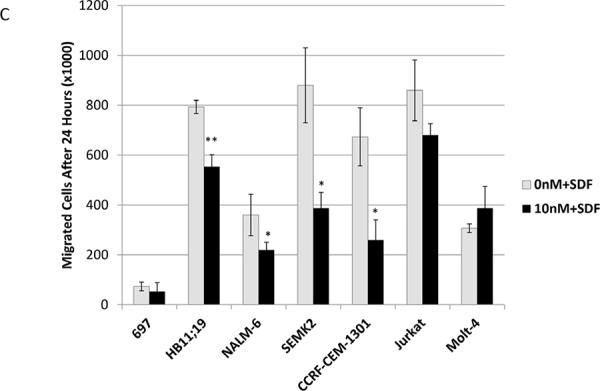
Treatment with POL5551 increases surface expression of CXCR7 and inhibits activation of CXCR4 via SDF-1α **A.** Surface CXCR7 expression in Nalm-6 and Jurkat after treatment with a concentration range of POL5551 over a 24-hour time course. The percentage of CXCR7-expressing cells at each concentration was normalized to the percentage of CXCR7-expressing cells in control-treated (0 nM) samples. **B.** ERK1/2 phosphorylation after stimulation with vehicle control or SDF-1α 75 ng/mL for 15 minutes. Results are depicted as a ratio of the MFI of cells stimulated with SDF-1α divided by the MFI of cells stimulated with vehicle control. **C.** Chemotaxis of cells through a permeable membrane toward SDF-1α 150 ng/mL after treatment with vehicle control or POL5551 10 nM. All experiments were performed independently and in duplicate. *p < 0.05, **p < 0.01 vs. 0 nM + SDF.

Next, we wanted to determine the functional effects of treatment with POL5551. Specifically, we hypothesized that CXCR4 antagonism with POL5551 would modulate expression of additional adhesion molecules and also inhibit activation of CXCR4 by SDF-1α. First, we measured surface expression of CXCR7 and VLA-4 after treatment with POL5551. CXCR7 is a second receptor for SDF-1α, [24, 25] while VLA-4 is an integrin, [26] and both are important in the interaction of leukemia cells with the bone marrow stromal microenvironment [27]. In addition, we have shown that treatment with plerixafor leads to an increase in surface expression of CXCR7 in ALL [10] and others have demonstrated that activation of CXCR4 leads to increased VLA-4-mediated adhesion of ALL blasts [4]. Therefore, we hypothesized that antagonism of CXCR4 with POL5551 would lead to increased surface expression of CXCR7 and VLA-4. We found that treatment of ALL cell lines with POL5551 did in fact lead to increased CXCR7 expression at early treatment time points that began to decrease by 24 hours (Figure 3A). We did not find a consistent effect of POL5551 on VLA- 4 (via anti-CD49d antibodies) surface expression (data not shown). Next, to determine if POL5551 could inhibit phosphorylation of a downstream target of CXCR4, we treated ALL cell lines with POL5551 or vehicle control and then stimulated CXCR4 with recombinant SDF-1α or vehicle control. Activation of CXCR4 with SDF-1α led to an increase in phosphorylation of ERK1/2 in control-treated cells. This increase in ERK1/2 phosphorylation was attenuated by increasing concentrations of POL5551 (Figure 3B). Finally, to determine if treatment with POL5551 could decrease SDF-1α-induced chemotaxis, we treated cells with POL5551 or vehicle control, and measured migration of ALL cells through a permeable membrane toward medium containing SDF-1α. Treatment with POL5551 led to a statistically significant decrease in chemotaxis in the majority of cell lines tested (Figure 3C). From these experiments, we concluded that antagonism of surface CXCR4 by POL5551 led to functional effects, including a transient increase in surface CXCR7 expression, decreased SDF-1α-induced phosphorylation of ERK1/2, and inhibition of SDF-1α-induced chemotaxis.

### POL5551 decreases stromal protection and increases sensitivity to chemotherapy

**Figure 4:**
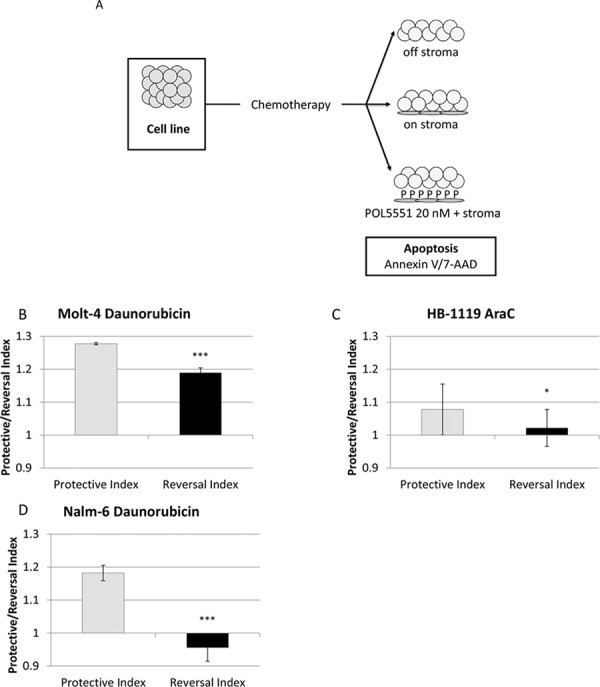
POL5551 enhances sensitivity to chemotherapy in a stromal co-culture model **A.** Treatment schema: cells were cultured off stroma, on normal human bone marrow stroma, or on stroma with POL5551 and treated with a concentration range of chemotherapy for 24 hours. Protective Index (PI) and Reversal Index (RI) after treatment **B.** with daunorubicin in MOLT-4, **C.** AraC in HB-1119, and **D.** daunorubicin in Nalm-6. *p < 0.05, ***p < 0.001 PI vs. RI.

We also hypothesized that POL5551 could decrease stromal protection from chemotherapy through antagonism of CXCR4. To investigate this hypothesis, we treated cells with a concentration range of chemotherapy in 3 culture conditions (Figure 4A). After chemotherapy treatment, we measured apoptosis with Annexin V and 7-AAD and calculated IC values by culture condition. We then used these IC values to calculate a Protective Index (PI), which quantifies the protective effect of stroma, and a Reversal Index (RI), which quantifies the ability of POL5551 to decrease or reverse stromal protection, as we have previously published [9, 10]. Briefly, we defined PI as the IC values on stroma divided by the IC values off stroma. Therefore, PI > 1 denotes stromal protection. Similarly, we defined RI as the IC values on stroma + POL5551 divided by the IC values off stroma. Therefore, RI > 1 signifies some stromal protection in the presence of POL5551 and stroma, RI < PI denotes a decrease in stromal protection by POL5551, and RI < 1 indicates complete reversal of stromal protection by POL5551. Stroma protected Molt- 4, HB-1119, and Nalm-6 from chemotherapy-induced apoptosis. Remarkably, treatment with 20 nM POL5551 was sufficient to decrease stromal protection in Molt-4 and HB-1119 and even reverse stromal protection in Nalm- 6 (Figures 4B–4D). These findings suggest that stromal protection from chemotherapy-induced apoptosis in ALL is mediated through CXCR4 and that administration of POL5551 decreases stromal protection and restores sensitivity to chemotherapy in our co-culture model.

### POL5551 antagonizes surface CXCR4 in primary samples of pediatric ALL

**Figure 5:**
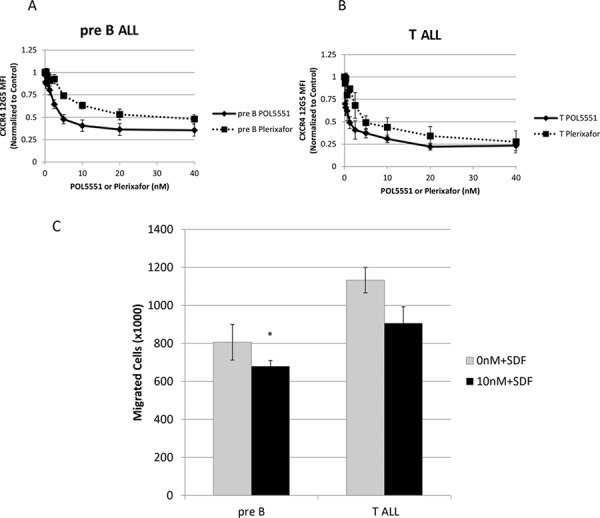
POL5551 inhibits 12G5 anti-CXCR4 antibody binding and SDF-1α-induced chemotaxis in primary samples of pediatric ALL Primary samples (*n* = 3 pre-B ALL, *n* = 3 T ALL) were treated with a concentration range of POL5551 and plerixafor. Cells were harvested for FACS after 2 hours of treatment and MFI were normalized to control. Average results after independent treatment of **A.** pre-B ALL primary samples (*n* = 3) and **B.** T ALL primary samples (*n* = 3). **C.** Chemotaxis of primary samples toward SDF-1α 150 ng/mL after treatment with vehicle control or POL5551 10 nM. p < 0.05 vs. 0nM+SDF.

We also wanted to verify some of our findings using primary samples of pediatric pre-B and T cell ALL. First, we treated primary samples of pediatric ALL with POL5551 and plerixafor and measured 12G5 antibody binding. We found that POL5551 potently blocked binding of the 12G5 antibody and was again significantly more potent than plerixafor in both pre-B (Figure 5A) and T cell ALL primary samples (Figure 5B). Next, we measured SDF-1α-induced chemotaxis as a measure of functional CXCR4 antagonism. Similar to our cell line experiments, we found that treatment with POL5551 decreased migration of primary ALL samples toward an SDF-1α gradient (Figure 5C). These data indicate that POL5551 is active against CXCR4 in primary samples of pediatric ALL.

### POL5551 increases sensitivity to cytarabine in an *in vivo* model of high-risk pediatric ALL

**Figure 6:**
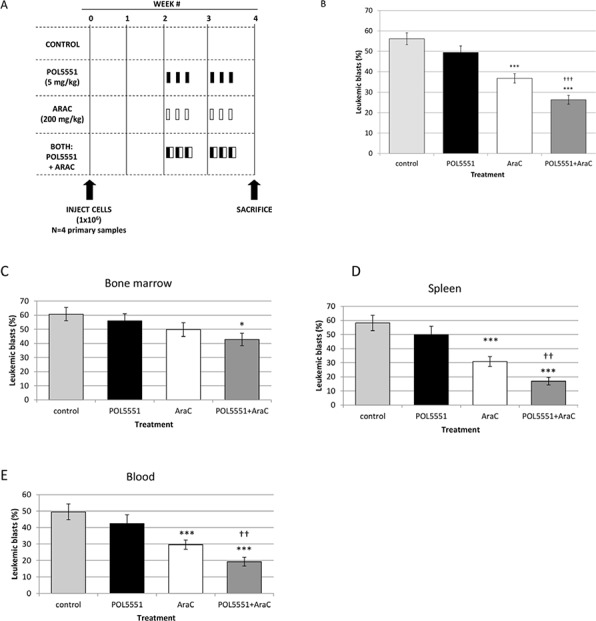
*In vivo* inhibition of CXCR4 with POL5551 sensitizes infant *MLL*-R ALL blasts to cytarabine **A.** Treatment schema: starting at week 2, mice were administered vehicle control, POL5551, cytarabine (AraC), or POL5551 in combination with AraC once daily on three consecutive days for 2 weeks (*n* = 5 mice/treatment cohort). Mice were sacrificed at the start of week 4, or 4 days after the last treatment. **B.** Overall leukemic burden: the results of 4 primary sample experiments were pooled. Overall leukemic burden was quantified by averaging the percentage of blasts (human CD45+ and CD19+) detected in the bone marrow, spleen, and peripheral blood. Quantification of leukemic blasts was performed in duplicate. Leukemic burden in **C.** bone marrow, **D.** spleen, and **E.** peripheral blood. *p < 0.05, **p < 0.01, ***p < 0.001 vs. control. ^†^*p* < 0.05, ^††^*p* < 0.01, ^†††^*p* < 0.001 vs. AraC.

Next, we created an *in vivo* xenograft model of an aggressive pediatric ALL to demonstrate that POL5551 can increase sensitivity to chemotherapy even in high-risk pediatric ALL. Therefore, we transplanted primary samples from infants with *MLL-*R ALL. These patients have an extremely poor prognosis [28] and primary samples of infant *MLL-*R ALL have engrafted very well in our previous experiments [8, 10]. To make our xenograft model even more aggressive, we transplanted cells that had already been passaged once through NOD/SCID/γ_c_^null^ (NSG) mice in order to select for leukemia-initiating cells from the cryopreserved primary samples. We performed this experiment using 4 different infant *MLL-*R ALL primary samples (Figure 6A). Overall leukemic burden (Figure 6B and Supplemental Figure 1A) did not differ significantly between mice treated with either vehicle control (56.2%) or POL5551 alone (49.5%). However, treatment with cytarabine (36.7%) or the combination of POL5551 and cytarabine (26.3%) significantly decreased total leukemic burden compared to vehicle control. Impressively, treatment with POL5551 and cytarabine significantly decreased total leukemic burden compared to treatment with cytarabine alone (*p* = 0.001), demonstrating that POL5551 increased sensitivity to cytarabine. When we analyzed leukemic burden by organ, we found a striking decrease in leukemic burden in mice treated with POL5551 and cytarabine compared to vehicle control (Figures 6C–6E and Supplemental Figures 1B–1D). Treatment with POL5551 and cytarabine also decreased leukemic burden compared to cytarabine alone in the bone marrow (42.8% vs. 49.5%), spleen (16.9 vs. 30.8%), and blood (19.3% vs. 29.6%). These results suggest that inhibition of CXCR4 with POL5551 may enhance sensitivity to cytarabine in infants with *MLL-*R ALL.

### *In vivo* anti-leukemic treatment modulates surface expression of CXCR4, CXCR7, and CD49d

Finally, we measured surface expression of CXCR4, CXCR7, and CD49d in leukemic blasts to determine if either the organ from which the blast was isolated or the treatment received had an effect on surface expression of these adhesion molecules. We found that surface expression of CXCR4 as measured by either the 12G5 or 1D9 antibody was lowest in the bone marrow and highest in the peripheral blood (Supplemental Figure 2A). Conversely, surface expression of CD49d was highest in the bone marrow and lowest in the peripheral blood (Supplemental Figure 2A). These findings are consistent with an inverse, homeostatic relationship between CXCR4 and VLA-4 that has been described previously [4, 10]. Interestingly, we also found that surface expression of CXCR7 was higher in the spleen and peripheral blood than in the bone marrow where the concentration of SDF-1α is the highest (Supplemental Figure 2A). These findings may indicate that leukemic blasts located outside of the bone marrow may have increased surface expression of CXCR4 and CXCR7 in order to become more responsive to SDF- 1α and home to areas of high SDF-1α concentration.

When we analyzed our results by treatment cohort, both cytarabine (*p* < 0.01 vs. control) and POL5551 and cytarabine (*p* < 0.05 vs. control) led to significantly decreased 12G5 antibody binding in splenic blasts with a moderate but non-significant decrease in 12G5 antibody binding in blasts isolated from bone marrow and peripheral blood (Supplemental Figure 2B). In contrast, a trend toward increased 1D9 antibody binding was found in blasts isolated from bone marrow and peripheral blood (Supplemental Figure 2C). Further, CXCR7 surface expression in leukemic blasts isolated from the bone marrow (*p* ≤ 0.01), spleen (*p* < 0.05), and peripheral blood (*p* ≤ 0.001) was significantly higher in mice treated with cytarabine-containing regimens, compared to control-treated mice (Supplemental Figure 2D). CD49d mean fluorescence index (MFI) was increased in leukemic blasts isolated from the bone marrow, spleen, and peripheral blood of mice treated with either cytarabine or POL5551 and cytarabine compared to control, but these differences were not statistically significant (Supplemental Figure 2E). Our data suggest that anti-leukemic therapies may alter the interaction of surviving blasts with the bone marrow microenvironment.

## DISCUSSION

Efforts to improve outcomes in pediatric ALL have largely been focused on factors intrinsic to the leukemic blast, such as genetic mutations or alterations in gene expression [2]. However, it is likely that survival signals from the bone marrow microenvironment also contribute to treatment failure and relapse [27, 29]. As such, many groups have studied CXCR4 antagonism as a means to improve outcomes in acute leukemias. Several preclinical studies have demonstrated that CXCR4 antagonism with plerixafor enhances *in vivo* sensitivity to anti-leukemic therapies [8, 10, 15]. Our paper is the first demonstration of the efficacy of the novel CXCR4 antagonist POL5551 in hematologic malignancies. Our experiments also show that POL5551 is a more potent antagonist of CXCR4 in pediatric ALL than plerixafor.

In our initial *in vitro* experiments, we found that POL5551 binds to surface CXCR4 at the 12G5- (and thus SDF-1α-) binding site, which results in functional consequences, namely the attenuation of SDF-1α-mediated phosphorylation of ERK1/2, inhibition of SDF-1α-induced chemotaxis, and restoration of chemosensitivity in a stroma co-culture model. We used infant *MLL-*R ALL primary samples to confirm these findings in an *in vivo* model. In previous work, we showed that *MLL-*R ALL primary samples have a survival advantage on bone marrow stroma feeder layers compared to non-*MLL-*R primary samples, which suggests that interaction with stroma is important in *MLL-*R ALL [8]. We selected cytarabine as our chemotherapeutic agent because of its tolerability in this mouse strain and because of its efficacy in infant ALL [30]. As expected, cytarabine decreased leukemic burden in our xenografts. Notably, the addition of POL5551 enhanced the efficacy of cytarabine; this was particularly evident in the bone marrow, where our dose of cytarabine did not lead to a significant decrease in leukemic burden compared to vehicle control. Collectively, these findings suggest that inhibition of leukemia-stromal interactions is an important component in the treatment of this high-risk subtype of pediatric ALL. There is also clinical evidence to support our hypothesis. The Interfant-99 trial, which enrolled 482 infants with ALL, reported that while 94% of patients achieved remission after induction therapy, only 47% achieved a long-term remission [28]. Further, patients who were negative for minimal residual disease (MRD) after induction and consolidation therapy still had a 5-year relapse rate of 13% [31]. In comparison, analysis of the most recent St. Jude Total Therapy trials reported a 5-year cumulative risk of relapse of only 5% in MRD-negative patients [32]. It is important to note both that the St. Jude MRD cutoff was 10-fold higher than Interfant-99 (0.001% vs. 0.0001% leukemia by PCR) and that only 2 of the 379 patients included in the St. Jude analysis were infants. These results suggest that leukemia-initiating cells are able to persist in infant ALL despite the achievement of a deep MRD-negative remission. Therefore, the use of CXCR4 antagonists may be a means to improve outcome in infant *MLL-*R ALL by targeting MRD and leukemia-initiating cells (LIC).

CXCR4 antagonism has been advanced clinically in acute leukemia. Trials of plerixafor as a chemosensitizing agent have been completed in adults with relapsed/refractory [21] and newly-diagnosed AML, [33] and children with relapsed/refractory acute leukemias [34]. One concern about CXCR4 antagonism is that mobilized hematopoietic stem cells will become more susceptible to chemotherapy and lead to delayed blood count recovery. These phase 1/2 trials showed tolerability and efficacy of plerixafor as a chemosensitizing agent and none of them reported prolonged count recovery. With regards to our experiments, POL5551 was recently shown to be an effective mobilizer of hematopoietic stem and progenitor cells in a mouse model, [20] and so we paid special attention to the health of our mice. We noted that mice in the cytarabine only cohorts were the only mice that were thin and hunched at sacrifice. Mice in the control cohort appeared well despite high leukemic burden. Those treated with POL5551 alone or in combination with cytarabine also looked healthy, suggesting that the combination did not have an adverse effect on the overall health of the mice. Another concern about CXCR4 antagonism as a treatment strategy in hematologic malignancies is the prospect of mobilizing leukemic cells from the bone marrow to other sites and contributing to disease spread. In our model, leukemic burden in mice treated with POL5551 alone was not significantly different than that of control-treated mice, suggesting that POL5551-mobilized blasts did not lead to an increase in disease. Further, none of the aforementioned trials reported the development of extramedullary disease after treatment.

CXCR4 antagonism not only enhances the efficacy of chemotherapy, but also of targeted agents, including FLT3 inhibitors, [8] and BCR-ABL inhibitors [17, 35]. Therefore, combining POL5551 with newer targeted agents, such as inhibitors of BRD4 [36] or DOT-1L [37] in *MLL-*R leukemias and phosphoinositide 3-kinase (PI3K) and mTOR inhibitors in hypodiploid ALL [38] and Philadelphia chromosome-like ALL, [39] may improve outcome in these high-risk pediatric leukemias. Our findings support the continued development of bone marrow microenvironment-targeted agents as a therapeutic strategy for pediatric ALL.

## MATERIALS AND METHODS

### Cell culture

Pre-B ALL cell lines (697, HB-1119, Nalm-6, SEMK2) and T cell ALL cell lines (CCRF-CEM-1301, Jurkat, Molt-4) were purchased from ATCC (Manassas, VA), DSMZ (Braunschweig, Germany), and Sigma-Aldrich (St. Louis, MO) and cultured in RPMI 1640 (Life Technologies, Grand Island, NY) with 10% fetal bovine serum (FBS, Gemini Bio-Products, West Sacramento, CA), penicillin-streptomycin (Life Technologies), and L-glutamine (Life Technologies). Diagnostic bone marrow or peripheral blood samples were collected under institutional review board (IRB)-approved protocols from newly diagnosed children with ALL. Cells were isolated, viably cryopreserved, and cultured as previously described [8–10]. Bone marrow was collected from adult bone marrow donors according to a Johns Hopkins IRB-approved protocol. Stromal cells were isolated and cultured as previously described [8–10].

### Fluorescence-activated cell sorting (FACS)

ALL cell lines and primary samples were treated with a concentration range of POL5551 (kindly provided by Polyphor, Allschwil, Switzerland) or plerixafor (kindly provided by Genzyme, Cambridge, MA). Cells were harvested at multiple time points, washed with ice-cold wash buffer (PBS with 0.5% bovine serum albumin, Sigma-Aldrich), stained with antibodies at manufacturer-recommended concentrations (12G5 anti-human CD184 [CXCR4]-APC, eBioscience, San Diego, CA; 12G5 anti-human CD184-PE-Cy5, BD Pharmingen, San Diego, CA; 1D9 anti-human CD184-PE, BD Pharmingen; 2B11 anti-mouse CD184-APC, eBioscience; anti-human CXCR7- PerCP, R&D Systems, Minneapolis, MN), washed, fixed with 1% paraformaldehyde (VWR, Radnor, PA), and stored at 4°C in the dark until analysis on a FACSCalibur (BD Biosciences, San Jose, CA). A live gate was drawn using FlowJo (TreeStar, Ashland, OR) according to forward/side scatter properties and used to calculate the MFI. MFI were normalized to the isotype control (IC) MFI at each time point and then normalized to the MFI:IC ratio of control-treated (0 nM) samples. Because CXCR7 MFI were relatively low, we quantified surface CXCR7 expression as the percentage of cells expressing CXCR7 (i.e., cells that had a CXCR7 MFI ≥ 95^th^ percentile of the IC MFI).

### PhosphoFACS

Cells were serum starved for 2 hours (RPMI with 0.5% FBS), treated with POL5551 or vehicle control for 1 hour, treated with recombinant SDF-1α (75 ng/mL, Peprotech, Rocky Hill, NJ) or vehicle control for 15 minutes, and prepared according to the BD Phosflow Protocol for Human PBMCs (BD Biosciences). Briefly, cells were fixed, permeabilized, stained with Alexa Fluor 488-conjugated anti-human ERK1/2 (pT202/pY204, BD Biosciences), and read on a FACSCalibur. The MFI of live cells were calculated using FlowJo and normalized to the IC MFI.

### Chemotaxis

Chemotaxis of cells treated with either vehicle control or 10 nM POL5551 toward medium with or without human recombinant SDF-1α (150 ng/mL) was measured as previously described [9, 10].

### Stroma co-culture

Cells were cultured in 3 conditions: off stroma, on stroma, or pretreated with 20 nM POL5551 for 30 minutes then plated on stroma. Cells were then treated with a concentration range of chemotherapy (cytarabine [AraC] or daunorubicin, Sigma-Aldrich) for 24 hours in duplicate wells. Concentration ranges of cytarabine and daunorubicin were unique to each cell line and contained the IC50 at 24 hours, based on pilot experiments. Cells were harvested, washed with ice-cold wash buffer, stained with anti-human CD19-FITC (pre-B cell lines) or anti-human CD3-FITC (T cell lines), washed, and stained with Annexin V-PE and 7-AAD (all antibodies from BD Pharmingen). After gating on CD19+/CD3+ cells, viable cells were defined as Annexin and 7-AAD negative. The IC10-IC90 values of each culture condition were calculated using Calcusyn (Biosoft, Cambridge, UK) and used to calculate the Protective Index (PI) and the Reversal Index (RI) [10].

### Xenograft experiments

Cryopreserved primary ALL cells were thawed, counted (TC10 Automated Cell Counter, BioRad, Hercules, CA), and resuspended in cold PBS. Adult NSG mice (Jackson Laboratories, Bar Harbor, ME) were sublethally irradiated (200 centiGray) 4 hours prior to the transplantation of 1 × 10^6^ viable cells via tail vein injection. Mice were started on Uniprim medicated feed (Harlan Laboratories, Frederick, MD) ≥ 24 hours prior to transplantation in order to decrease opportunistic infections. Peripheral blood was collected via cheek venipuncture 4 to 6 weeks post-transplant and analyzed by FACS after staining with anti-human CD19-FITC and anti-human CD45-PE (BD Pharmingen). Leukemic blasts were defined as cells that co-expressed human CD45 and human CD19. Mice were sacrificed 2 to 4 weeks post-engraftment and leukemic cells were harvested from the spleen and bone marrow.

Leukemic blasts (1 × 10^6^ per mouse) were then secondarily transplanted into sublethally irradiated NSG mice for treatment experiments. After a 2-week period of engraftment, as we had done in previous experiments [8, 10], mice were divided into 4 treatment cohorts (*n* = 5 mice/cohort) and treated on 3 consecutive days for 2 weeks with 1) vehicle control (PBS via subcutaneous and intraperitoneal injection), 2) POL5551 (5 mg/kg via subcutaneous injection), 3) cytarabine (200 mg/kg via intraperitoneal injection), or 4) POL5551 followed by cytarabine 4 hours later. After sacrifice, cells were isolated from bone marrow, spleen, and peripheral blood; counted; and stained with anti-human FACS antibodies (CD19- FITC, CD45-PE/APC, 12G5 CXCR4-APC, 1D9 CXCR4-PE, CXCR7-PerCP, and/or CD49d-PE-Cy5).

## SUPPLEMENTARY FIGURES


