# Supplementary material for: POL5551, a novel and potent CXCR4 antagonist, enhances sensitivity to chemotherapy in pediatric ALL
Source: Oncotarget. 2015 Sep 3;6(31):30902–18. doi: 10.18632/oncotarget.5094 (PMC4741576; doi:10.18632/oncotarget.5094)
Supplement: Supplementary file 1 [file oncotarget-06-30902-s001.pdf]

SUPPLEMENTARY FIGURES

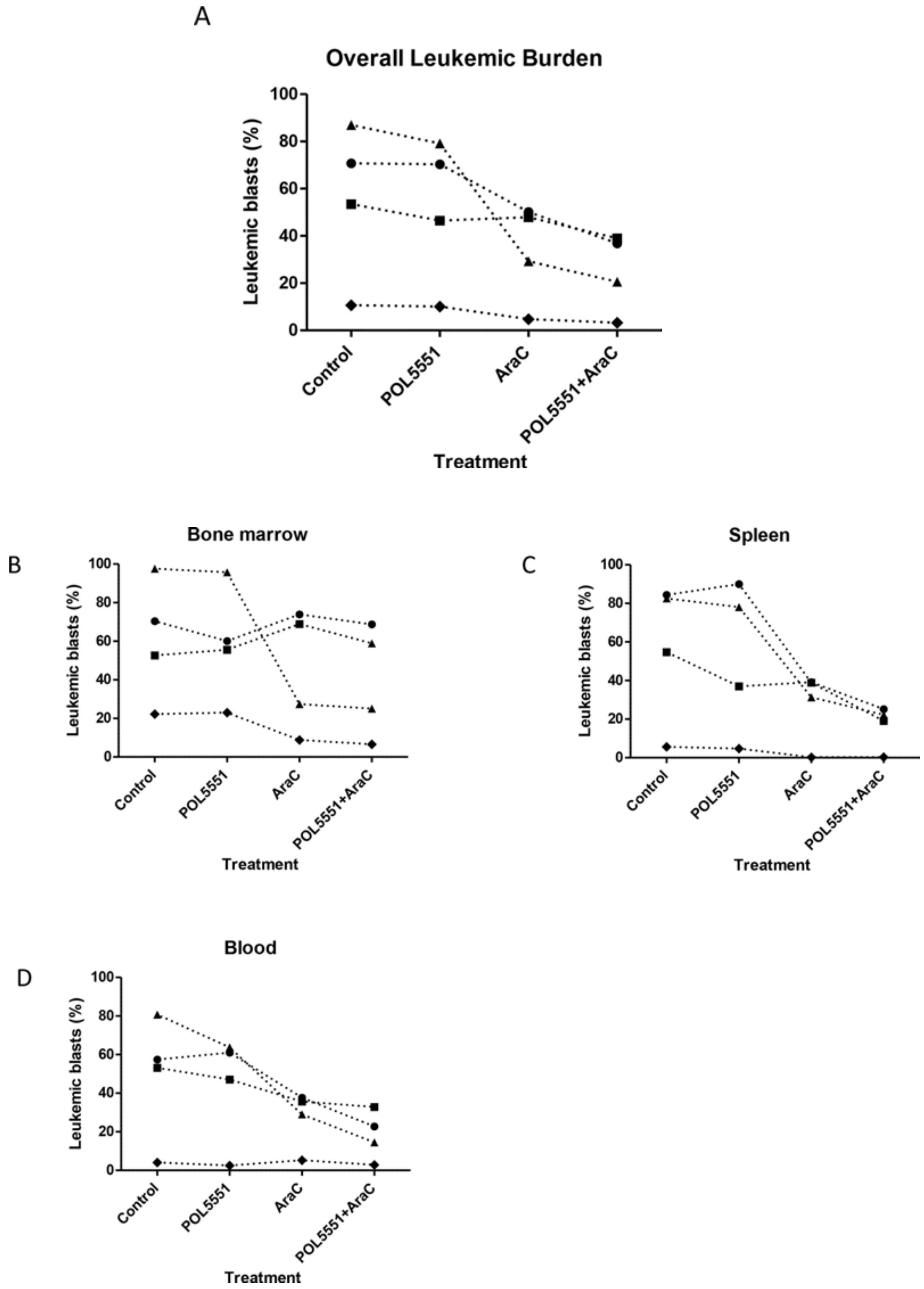

Supplementary Figure S1: *In vivo* inhibition of CXCR4 with POL5551 sensitizes infant MLL-R ALL blasts to cytarabine. A. Overall leukemic burden was quantified by averaging the percentage of blasts (human CD45+ and CD19+) detected in the bone marrow, spleen, and peripheral blood. Quantification of leukemic blasts was performed in duplicate. Each data point represents the mean leukemic burden for a single primary sample ($n = 5$ mice/treatment cohort). Each primary sample ($n = 4$) is represented by a unique shape and treatment cohorts for each primary sample are connected by dotted lines. Leukemic burden in **B.** bone marrow, **C.** spleen, and **D.** peripheral blood. $^{*}p < 0.05$, $^{**}p < 0.01$, $^{***}p < 0.001$ vs. control. $^{\dagger}p < 0.05$, $^{\dagger\dagger}p < 0.01$, $^{\dagger\dagger\dagger}p < 0.001$ vs. AraC.

A

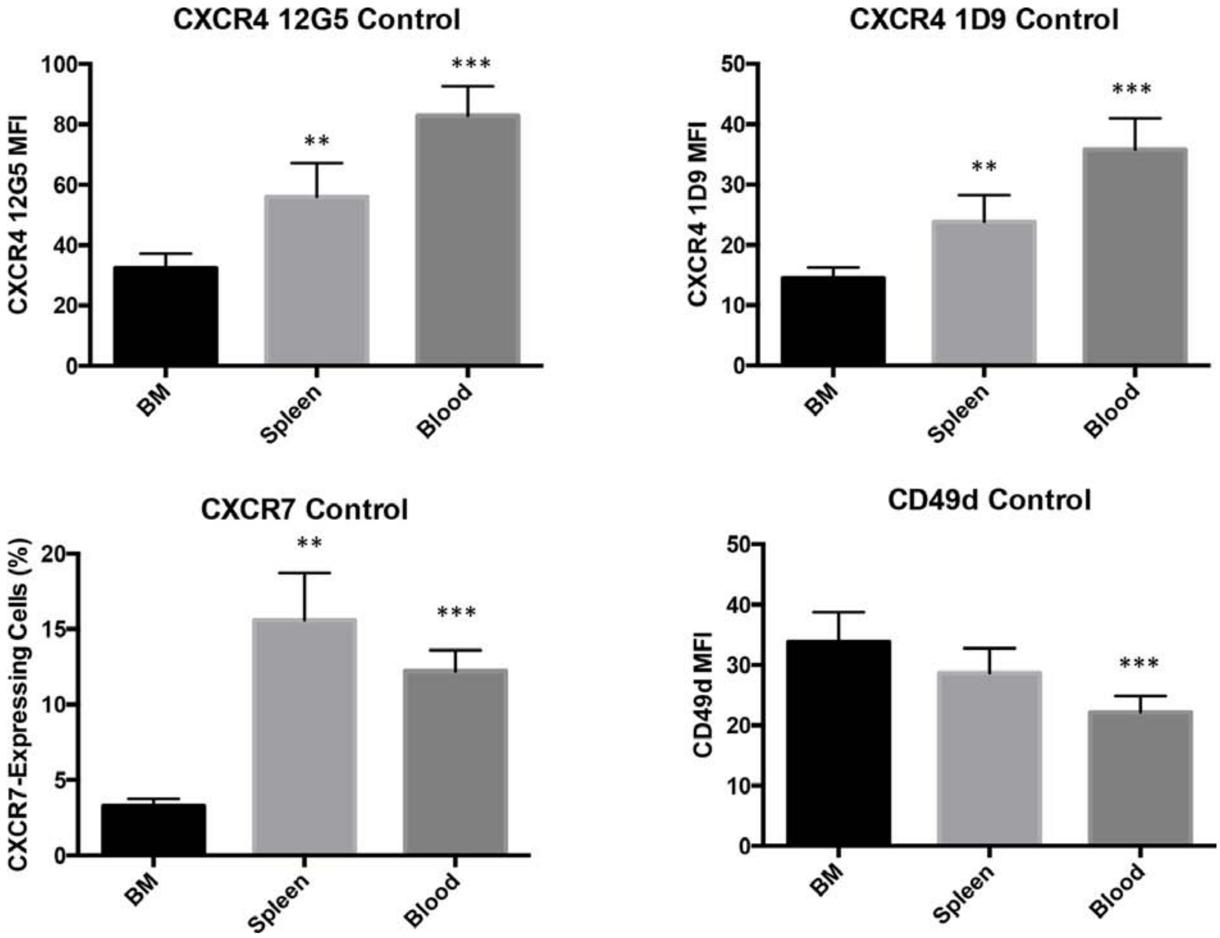

Supplementary Figure S2: Surface expression of CXCR4, CXCR7, and VLA-4 (CD49d) is affected by organ and treatment. Surface expression of CXCR4, CXCR7, and CD49d were measured by FACS in the leukemic blast populations (co-expressing human CD45+ and CD19+). The results of 4 primary sample experiments were pooled. Each data point represents the average MFI for a single primary sample. **A.** Surface expression of CXCR4 (12G5), CXCR4 (1D9), CXCR7, and CD49d by organ in control-treated mice. *$p < 0.05$, **$p < 0.01$, ***$p < 0.001$ vs. BM (bone marrow).

(*Continued*)

B

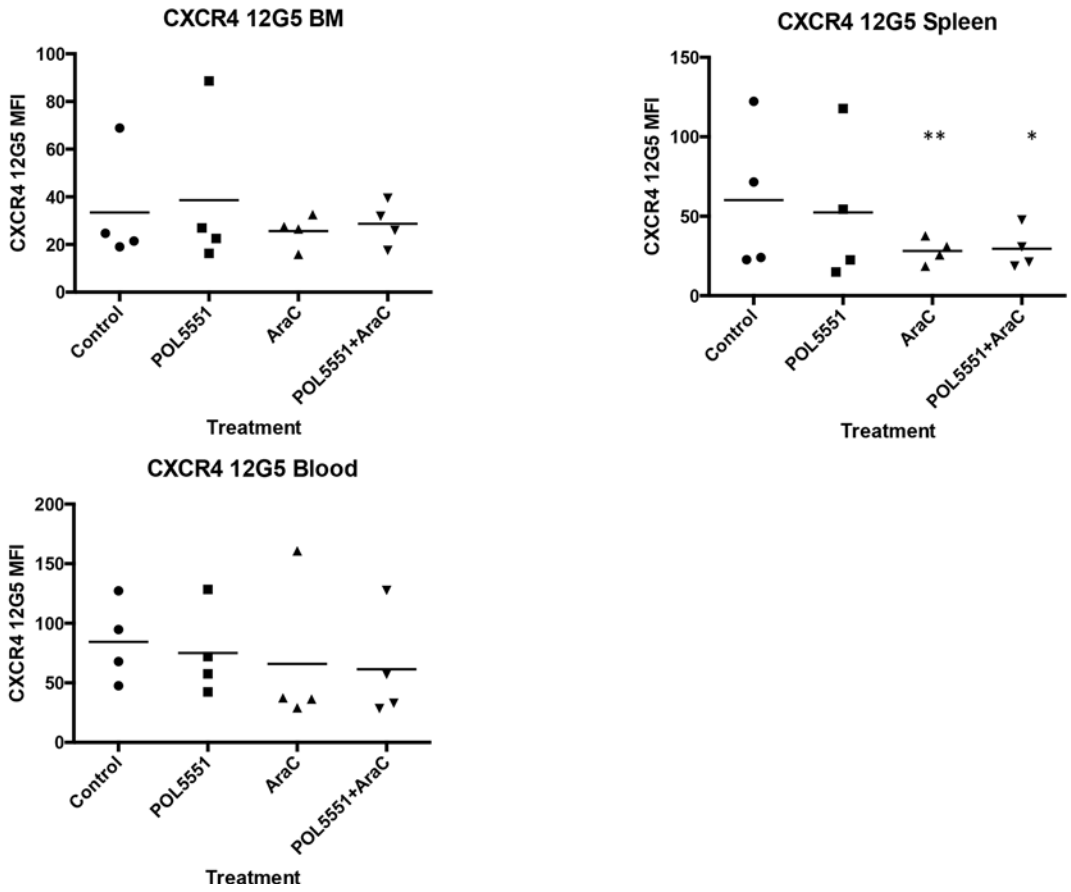

Supplementary Figure S2 (*Continued*): B. Surface expression of CXCR4 by 12G5 antibody binding by organ and treatment. *p < 0.05, **p < 0.01, ***p < 0.001 vs. control.

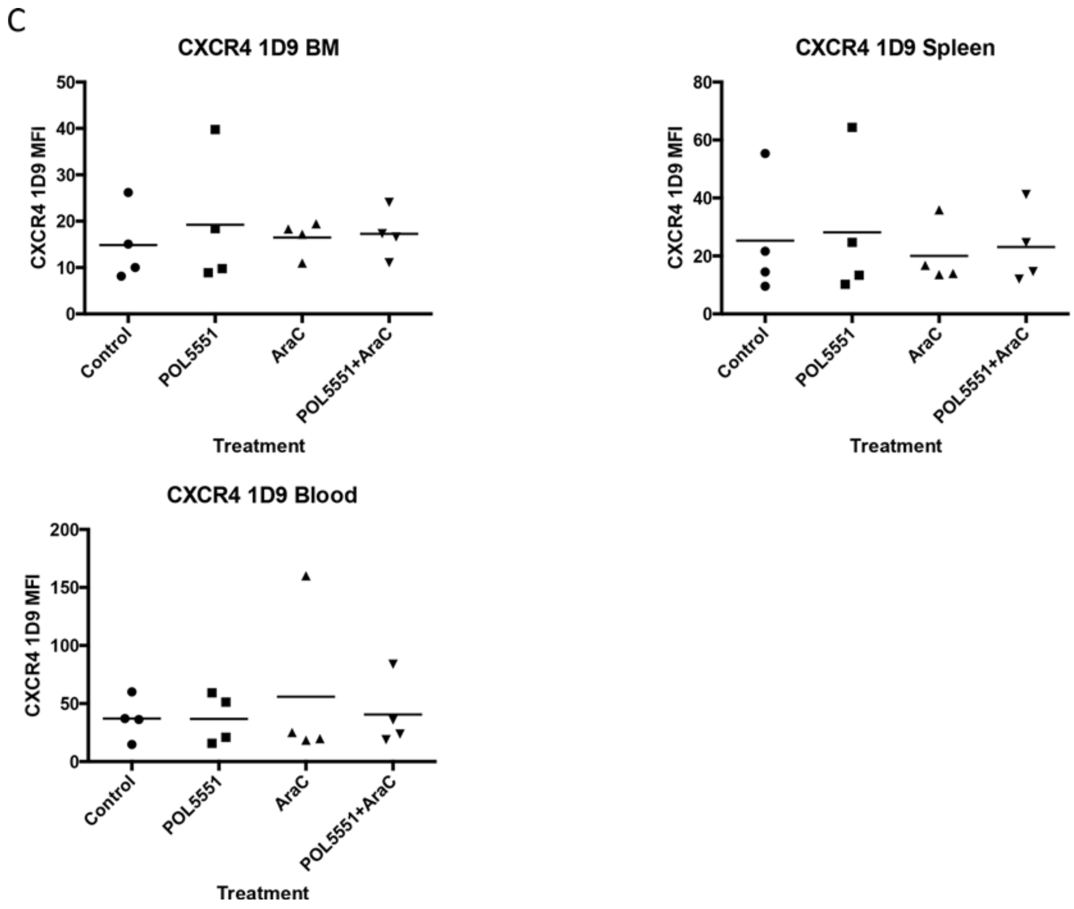

Supplementary Figure S2 (*Continued*): C. Surface expression of CXCR4 by 1D9 antibody binding by organ and treatment. *p < 0.05, **p < 0.01, ***p < 0.001 vs. control.

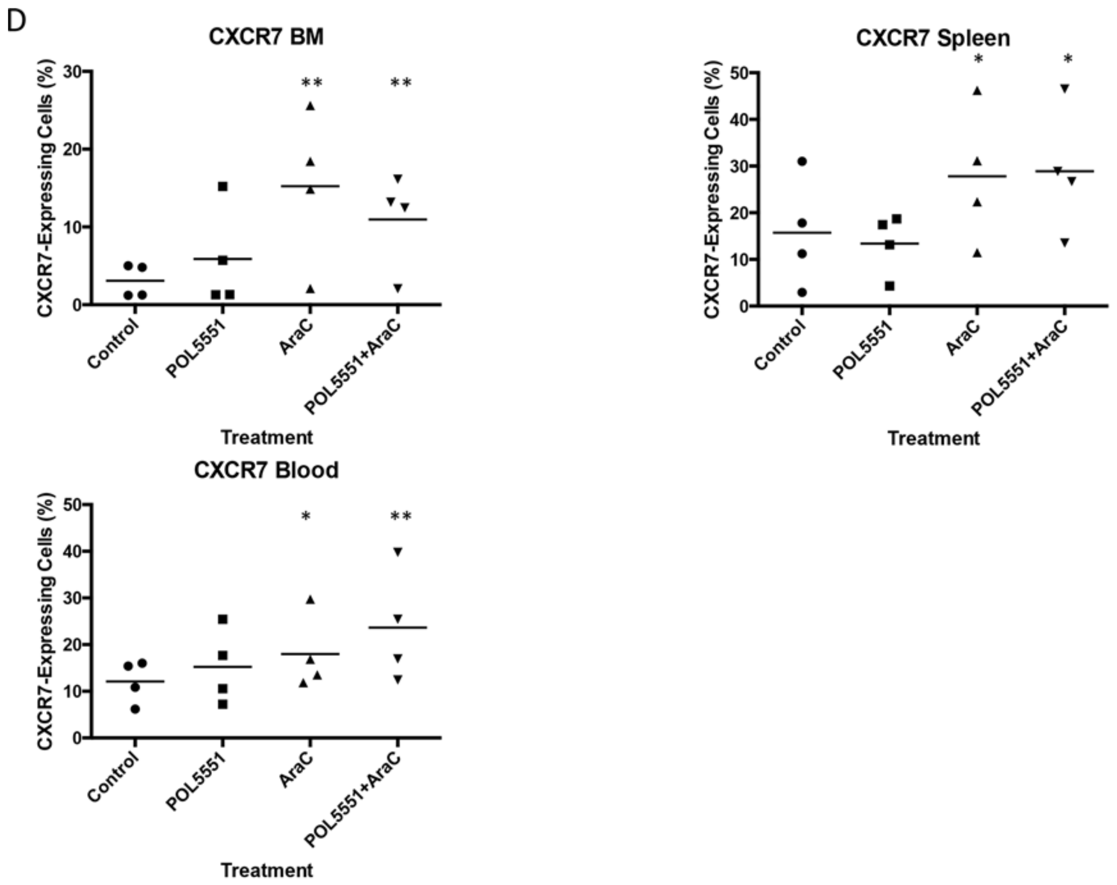

Supplementary Figure S2 (*Continued*):D. Surface expression of CXCR7 by organ and treatment. $*p < 0.05$, $**p < 0.01$, $***p < 0.001$ vs. control

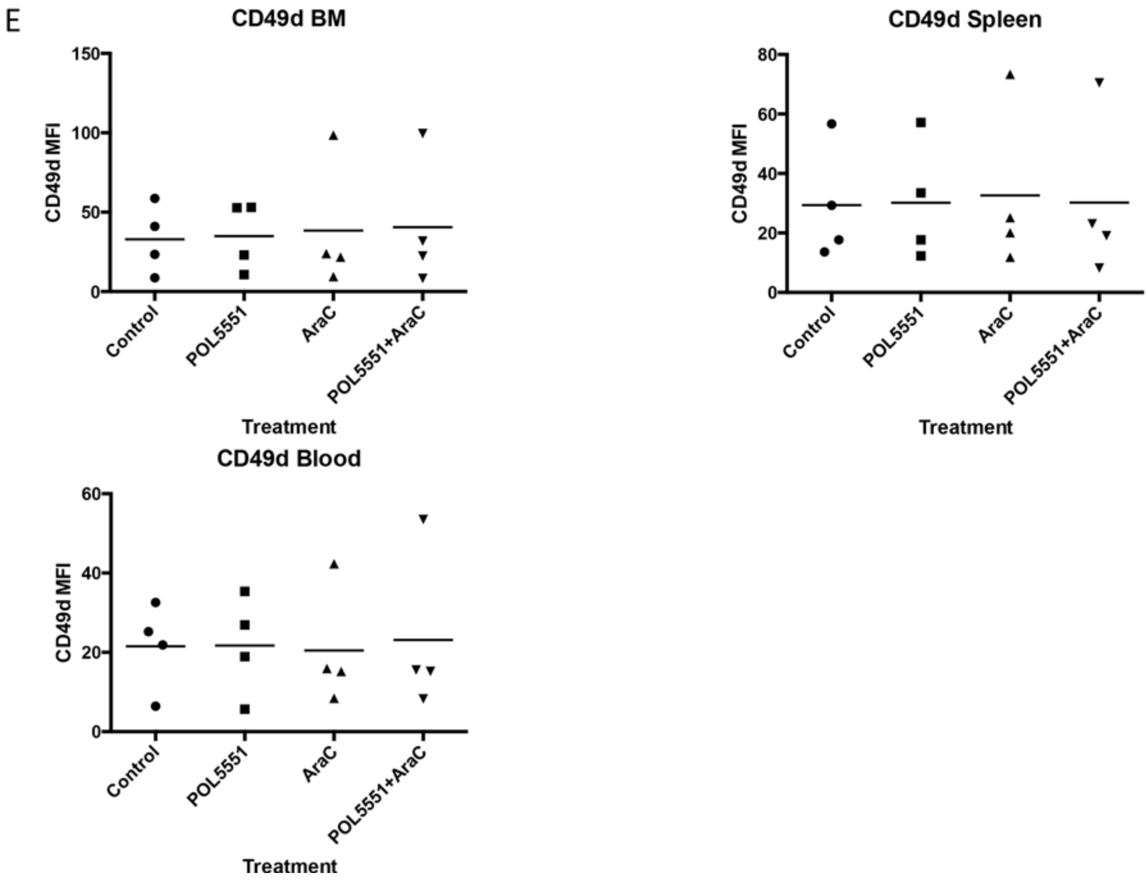

Supplementary Figure S2 (*Continued*): E. Surface expression of CD49d by organ and treatment. $*p < 0.05$, $**p < 0.01$, $***p < 0.001$ vs. control.
